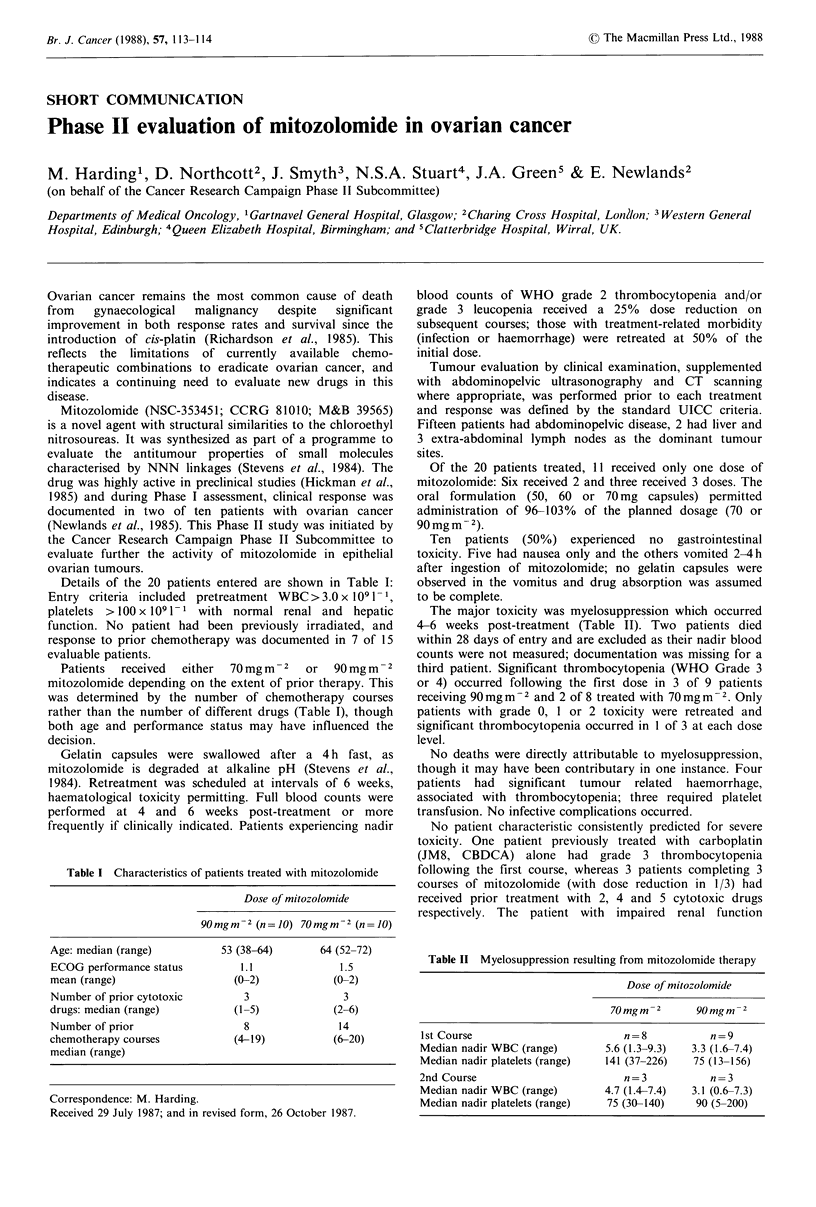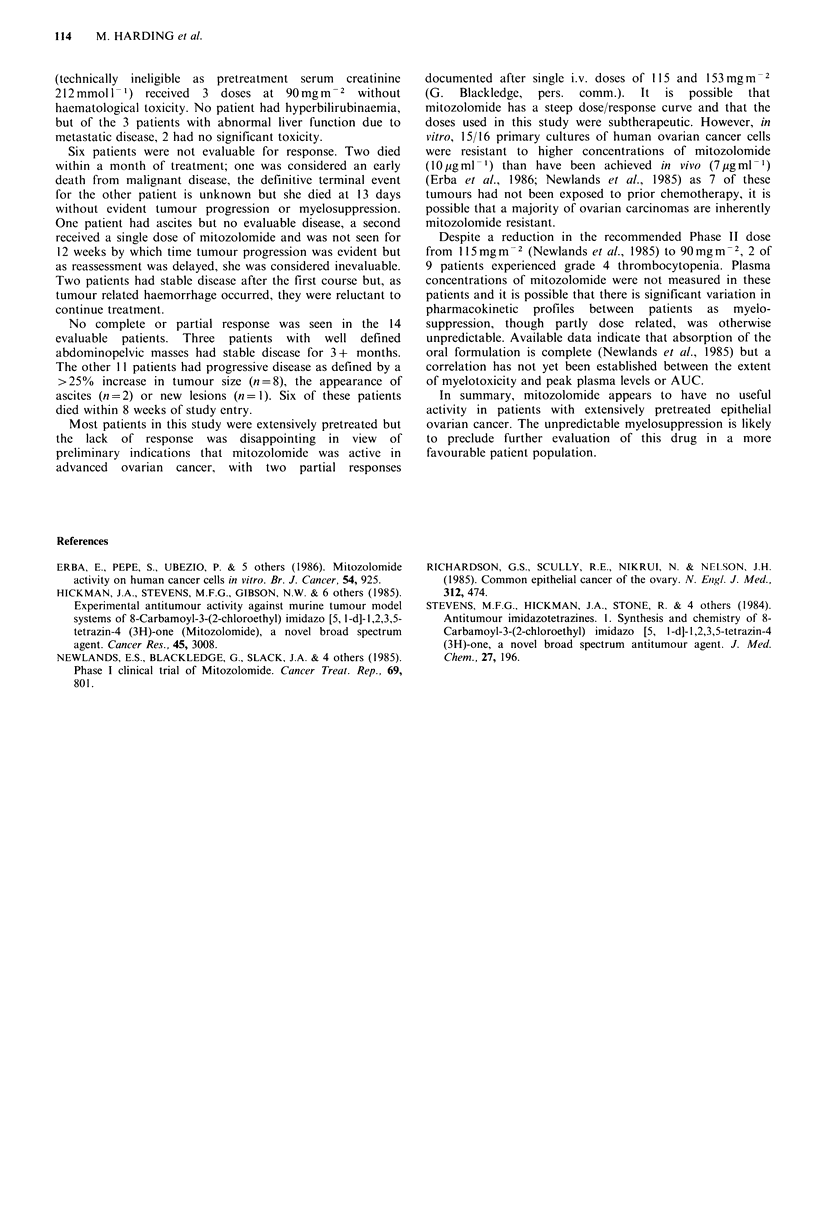# Phase II evaluation of mitozolomide in ovarian cancer.

**DOI:** 10.1038/bjc.1988.21

**Published:** 1988-01

**Authors:** M. Harding, D. Northcott, J. Smyth, N. S. Stuart, J. A. Green, E. Newlands

**Affiliations:** Department of Medical Oncology, Gartnavel General Hospital, Glasgow, UK.


Br. J. Cancer (1988), 57, 113–114 © The Macmillan Press Ltd., 1988

SHORT COMMUNICATION

### Phase II evaluation of mitozolomide in ovarian cancer

M. Harding[1], D. Northcott[2], J. Smyth[3], N.S.A. Stuart[4], J.A. Green[5] & E. Newlands[2]
(on behalf of the Cancer Research Campaign Phase II Subcommittee)

*Departments of Medical Oncology, [1]Gartnavel General Hospital, Glasgow; [2]Charing Cross Hospital, London; [3]Western General Hospital, Edinburgh; [4]Queen Elizabeth Hospital, Birmingham; and [5]Clatterbridge Hospital, Wirral, UK.*

Ovarian cancer remains the most common cause of death from gynaecological malignancy despite significant improvement in both response rates and survival since the introduction of *cis*-platin (Richardson *et al.*, 1985). This reflects the limitations of currently available chemotherapeutic combinations to eradicate ovarian cancer, and indicates a continuing need to evaluate new drugs in this disease.

Mitozolomide (NSC-353451; CCRG 81010; M&B 39565) is a novel agent with structural similarities to the chloroethyl nitrosoureas. It was synthesized as part of a programme to evaluate the antitumour properties of small molecules characterised by NNN linkages (Stevens *et al.*, 1984). The drug was highly active in preclinical studies (Hickman *et al.*, 1985) and during Phase I assessment, clinical response was documented in two of ten patients with ovarian cancer (Newlands *et al.*, 1985). This Phase II study was initiated by the Cancer Research Campaign Phase II Subcommittee to evaluate further the activity of mitozolomide in epithelial ovarian tumours.

Details of the 20 patients entered are shown in Table I: Entry criteria included pretreatment WBC$>3.0\times10^9\,l^{-1}$, platelets $>100\times10^9\,l^{-1}$ with normal renal and hepatic function. No patient had been previously irradiated, and response to prior chemotherapy was documented in 7 of 15 evaluable patients.

Patients received either $70\,mg\,m^{-2}$ or $90\,mg\,m^{-2}$ mitozolomide depending on the extent of prior therapy. This was determined by the number of chemotherapy courses rather than the number of different drugs (Table I), though both age and performance status may have influenced the decision.

Gelatin capsules were swallowed after a 4h fast, as mitozolomide is degraded at alkaline pH (Stevens *et al.*, 1984). Retreatment was scheduled at intervals of 6 weeks, haematological toxicity permitting. Full blood counts were performed at 4 and 6 weeks post-treatment or more frequently if clinically indicated. Patients experiencing nadir blood counts of WHO grade 2 thrombocytopenia and/or grade 3 leucopenia received a 25% dose reduction on subsequent courses; those with treatment-related morbidity (infection or haemorrhage) were retreated at 50% of the initial dose.

Tumour evaluation by clinical examination, supplemented with abdominopelvic ultrasonography and CT scanning where appropriate, was performed prior to each treatment and response was defined by the standard UICC criteria. Fifteen patients had abdominopelvic disease, 2 had liver and 3 extra-abdominal lymph nodes as the dominant tumour sites.

Of the 20 patients treated, 11 received only one dose of mitozolomide: Six received 2 and three received 3 doses. The oral formulation (50, 60 or 70 mg capsules) permitted administration of 96–103% of the planned dosage (70 or $90\,mg\,m^{-2}$).

Ten patients (50%) experienced no gastrointestinal toxicity. Five had nausea only and the others vomited 2–4 h after ingestion of mitozolomide; no gelatin capsules were observed in the vomitus and drug absorption was assumed to be complete.

The major toxicity was myelosuppression which occurred 4–6 weeks post-treatment (Table II). Two patients died within 28 days of entry and are excluded as their nadir blood counts were not measured; documentation was missing for a third patient. Significant thrombocytopenia (WHO Grade 3 or 4) occurred following the first dose in 3 of 9 patients receiving $90\,mg\,m^{-2}$ and 2 of 8 treated with $70\,mg\,m^{-2}$. Only patients with grade 0, 1 or 2 toxicity were retreated and significant thrombocytopenia occurred in 1 of 3 at each dose level.

No deaths were directly attributable to myelosuppression, though it may have been contributary in one instance. Four patients had significant tumour related haemorrhage, associated with thrombocytopenia; three required platelet transfusion. No infective complications occurred.

No patient characteristic consistently predicted for severe toxicity. One patient previously treated with carboplatin (JM8, CBDCA) alone had grade 3 thrombocytopenia following the first course, whereas 3 patients completing 3 courses of mitozolomide (with dose reduction in 1/3) had received prior treatment with 2, 4 and 5 cytotoxic drugs respectively. The patient with impaired renal function

**Table I** Characteristics of patients treated with mitozolomide

| | *Dose of mitozolomide* | |
|---|---|---|
| | *90 mg m$^{-2}$ (n=10)* | *70 mg m$^{-2}$ (n=10)* |
| Age: median (range) | 53 (38–64) | 64 (52–72) |
| ECOG performance status mean (range) | 1.1 (0–2) | 1.5 (0–2) |
| Number of prior cytotoxic drugs: median (range) | 3 (1–5) | 3 (2–6) |
| Number of prior chemotherapy courses median (range) | 8 (4–19) | 14 (6–20) |

Correspondence: M. Harding.
Received 29 July 1987; and in revised form, 26 October 1987.

**Table II** Myelosuppression resulting from mitozolomide therapy

| | *Dose of mitozolomide* | |
|---|---|---|
| | *70 mg m$^{-2}$* | *90 mg m$^{-2}$* |
| 1st Course | $n=8$ | $n=9$ |
| Median nadir WBC (range) | 5.6 (1.3–9.3) | 3.3 (1.6–7.4) |
| Median nadir platelets (range) | 141 (37–226) | 75 (13–156) |
| 2nd Course | $n=3$ | $n=3$ |
| Median nadir WBC (range) | 4.7 (1.4–7.4) | 3.1 (0.6–7.3) |
| Median nadir platelets (range) | 75 (30–140) | 90 (5–200) |

(technically ineligible as pretreatment serum creatinine 212 mmol l$^{-1}$) received 3 doses at 90 mg m$^{-2}$ without haematological toxicity. No patient had hyperbilirubinaemia, but of the 3 patients with abnormal liver function due to metastatic disease, 2 had no significant toxicity.

Six patients were not evaluable for response. Two died within a month of treatment; one was considered an early death from malignant disease, the definitive terminal event for the other patient is unknown but she died at 13 days without evident tumour progression or myelosuppression. One patient had ascites but no evaluable disease, a second received a single dose of mitozolomide and was not seen for 12 weeks by which time tumour progression was evident but as reassessment was delayed, she was considered inevaluable. Two patients had stable disease after the first course but, as tumour related haemorrhage occurred, they were reluctant to continue treatment.

No complete or partial response was seen in the 14 evaluable patients. Three patients with well defined abdominopelvic masses had stable disease for 3+ months. The other 11 patients had progressive disease as defined by a >25% increase in tumour size ($n=8$), the appearance of ascites ($n=2$) or new lesions ($n=1$). Six of these patients died within 8 weeks of study entry.

Most patients in this study were extensively pretreated but the lack of response was disappointing in view of preliminary indications that mitozolomide was active in advanced ovarian cancer, with two partial responses documented after single i.v. doses of 115 and 153 mg m$^{-2}$ (G. Blackledge, pers. comm.). It is possible that mitozolomide has a steep dose/response curve and that the doses used in this study were subtherapeutic. However, *in vitro*, 15/16 primary cultures of human ovarian cancer cells were resistant to higher concentrations of mitozolomide (10 $\mu$g ml$^{-1}$) than have been achieved *in vivo* (7 $\mu$g ml$^{-1}$) (Erba *et al.*, 1986; Newlands *et al.*, 1985) as 7 of these tumours had not been exposed to prior chemotherapy, it is possible that a majority of ovarian carcinomas are inherently mitozolomide resistant.

Despite a reduction in the recommended Phase II dose from 115 mg m$^{-2}$ (Newlands *et al.*, 1985) to 90 mg m$^{-2}$, 2 of 9 patients experienced grade 4 thrombocytopenia. Plasma concentrations of mitozolomide were not measured in these patients and it is possible that there is significant variation in pharmacokinetic profiles between patients as myelosuppression, though partly dose related, was otherwise unpredictable. Available data indicate that absorption of the oral formulation is complete (Newlands *et al.*, 1985) but a correlation has not yet been established between the extent of myelotoxicity and peak plasma levels or AUC.

In summary, mitozolomide appears to have no useful activity in patients with extensively pretreated epithelial ovarian cancer. The unpredictable myelosuppression is likely to preclude further evaluation of this drug in a more favourable patient population.

### References

ERBA, E., PEPE, S., UBEZIO, P. & 5 others (1986). Mitozolomide activity on human cancer cells *in vitro*. *Br. J. Cancer*, **54**, 925.

HICKMAN, J.A., STEVENS, M.F.G., GIBSON, N.W. & 6 others (1985). Experimental antitumour activity against murine tumour model systems of 8-Carbamoyl-3-(2-chloroethyl) imidazo [5, 1-d]-1,2,3,5-tetrazin-4 (3H)-one (Mitozolomide), a novel broad spectrum agent. *Cancer Res.*, **45**, 3008.

NEWLANDS, E.S., BLACKLEDGE, G., SLACK, J.A. & 4 others (1985). Phase I clinical trial of Mitozolomide. *Cancer Treat. Rep.*, **69**, 801.

RICHARDSON, G.S., SCULLY, R.E., NIKRUI, N. & NELSON, J.H. (1985). Common epithelial cancer of the ovary. *N. Engl. J. Med.*, **312**, 474.

STEVENS, M.F.G., HICKMAN, J.A., STONE, R. & 4 others (1984). Antitumour imidazotetrazines. 1. Synthesis and chemistry of 8-Carbamoyl-3-(2-chloroethyl) imidazo [5, 1-d]-1,2,3,5-tetrazin-4 (3H)-one, a novel broad spectrum antitumour agent. *J. Med. Chem.*, **27**, 196.